# Finite Element Analysis of porously punched prosthetic short stem virtually designed for simulative uncemented Hip Arthroplasty

**DOI:** 10.1186/s12891-017-1651-9

**Published:** 2017-07-11

**Authors:** Matthew Jian-Qiao Peng, Hai-Yan Chen, Yong Hu, XiangYang Ju, Bo Bai

**Affiliations:** 1 Guangdong Orthopedics Implantation key Lab, Orthopedics Department of 1st Affiliated Hospital, Guangzhou Medical University, 151 YanJiangXi Rd, Guangzhou, 510120 China; 2 Neural Electrophysiology Lab, University of Hong Kong, Room 501, Haking Wong Building, Pokfulam Road, Pok Fu Lam, Hong Kong; 3 Clinical Physics & Bioengineering Department, University of Glasgow, 378 Sauchiehall St., Glasgow, G2 3JZ UK

**Keywords:** Uncemented short stem, Porously punched prosthesis, Artificial joint replacement, Contact stress, Finite Element Analysis

## Abstract

**Background:**

There is no universal hip implant suitably fills all femoral types, whether prostheses of porous short-stem suitable for Hip Arthroplasty is to be measured scientifically.

**Methods:**

Ten specimens of femurs scanned by CT were input onto Mimics to rebuild 3D models; their *stl format dataset were imported into Geomagic-Studio for simulative osteotomy; the generated *.igs dataset were interacted by UG to fit solid models; the prosthesis were obtained by the same way from patients, and bored by punching bears designed by Pro-E virtually; cements between femora and prosthesis were extracted by deleting prosthesis; in HyperMesh, all compartments were assembled onto four artificial joint style as: (a) cemented long-stem prosthesis; (b) porous long-stem prosthesis; (c) cemented short-stem prosthesis; (d) porous short-stem prosthesis. Then, these numerical models of Finite Element Analysis were exported to AnSys for numerical solution.

**Results:**

Observed whatever from femur or prosthesis or combinational femora-prostheses, “Kruskal-Wallis” value *p* > 0.05 demonstrates that displacement of (d) ≈ (a) ≈ (b) ≈ (c) shows nothing different significantly by comparison with 600 N load. If stresses are tested upon prosthesis, (d) ≈ (a) ≈ (b) ≈ (c) is also displayed; if upon femora, (d) ≈ (a) ≈ (b) < (c) is suggested; if upon integral joint, (d) ≈ (a) < (b) < (c) is presented.

**Conclusions:**

Mechanically, these four sorts of artificial joint replacement are stabilized in quantity. Cemented short-stem prostheses present the biggest stress, while porous short-stem & cemented long-stem designs are equivalently better than porous long-stem prostheses and alternatives for femoral-head replacement. The preferred design of those two depends on clinical conditions. The cemented long-stem is favorable for inactive elders with osteoporosis, and porously punched cementless short-stem design is suitable for patients with osteoporosis, while the porously punched cementless short-stem is favorable for those with a cement allergy. Clinically, the strength of this study is to enable preoperative strategy to provide acute correction and decrease procedure time.

## Background

A meticulous preoperative strategy is of paramount importance before performing a complex operation that could employ multiple available reconstructive techniques. Additionally, proper modular implants of artificial prostheses in terms of type and length must be selected prior to hip surgery. There are various procedures for treating femoral neck fractures. For fresh fracture without dislocation, internal fixation is preferable. For elderly patients with osteoarthritis or rheumatoid arthritis, Hip Arthroplasty (HA) is a prevalent approach to achieve function restoration of degenerative joint diseases in the twentieth century. However, there is no universal hip implant to suitably treat all femoral types, and appropriate design is demanded that could prevent complications from the implant’s geometric mismatch. The previous literature indicates that joint fracture is quite likely to occur if there are high stresses in the fixation areas [1, 2], and conventional stems have showed disadvantages, such as proximal stress shielding, loss of bone stock, and a risk of fracture [3]. Dennis et al. suggests that cement carries a risk of fatigue failure than bone since more cracks were formed in cement than in bone in this study [4]. Fawzi et al. posited that bone cement material, stem material and shape significantly affect the Total Hip Joint performance, as well as that the stem length has significant effects on resultant Von Mises stresses for bone, stem and cement [5]. Short-stem prostheses were reported primarily to preserve femoral bone stock, reduce the amount of osteotomy during femoral preparation and facilitate future revision surgery possibly [6], but there has been doubt that they can maintain stability, osseointegration and survival of the femoral stem [5]. Walker et al. suggests that the femoral moment of the compressive strain of an uncemented prosthesis is only 30% of that for cemented prostheses [7]. Whiteside et al. declared that cementless short-stem prostheses preserve femoral neck with greater torsional stability while reducing distal migration of the stem [8]. Because uncemented straight stems have demonstrated excellent long-term results into the third decade, we hypothesize that the stem type or size may affect the value and distribution of mechanical stress, but newly designed short-stem designs need to be critically evaluated. The issues of “Long or short stem?” & “Cementlss or cement stem?” will therefore be investigated in this study. To realize this aim, first of all, a three-dimensional (3D) femoral model for preoperative planning is achieved by Reverse Engineering Software that mimicked operative protocol virtually; then, four sorts of simulative stems (Cemented stem of long and short without pore, Cementless stem of long and short with pore) were designed and compared by Finite Element (FE) method mechanically. Finally, clinical contention concerning the bio-mechanism is discussed in the conclusion.

## Methods

### Experimental material

The facilities needed are listed as: 10 sets of specimens (female patients aged 20 ~ 40 yrs) of femora with prostheses from our hospital (in accordance with standards of Guangzhou Medical University Committee (#2014A020215035) and with the 1964 Helsinki declaration) scanned by Computed Tomography **(**CT, Toshiba, Japan), medical Image Processor Mimics (Materialise, Leuvan, Belgium), Reverse Engineering software Geomagic-Studio-12 (3D Systems, Rock Hill, USA), interactive Computer Aided Design (CAD) / Computer Aided Manufacturing (CAM) package UG-8.0 (Siemens, Germany), 3D drawing processor ProE/Engineer-5 (Parametric Tech Corp, USA), and Finite Element Analysis (FEA) package AnSys-14 (ANSYS, Inc. Pennsylvania, USA), pre-FEA processor Hypermesh-13 (Altair Engineering, USA), and Universal Testing Machine (UTM, Zwick Roell, Germany).

### Experimental procedure

#### Reconstruction of the femoral model

Ten femora scanned by CT in Dicom format were exported to Mimics. The default bony gray value range of 226 ~ 2311 was set to the threshold so that the femur was separated from the proximal femur extracted by “Region Growing”. The “Edit mask” function on the “Mask” module was then executed to erasure / protract / calculate the 3D model [9], and a proximal femur model of the *.stl format was developed for accurate measurement.

#### Distal femur developed

**Fig. 1:**
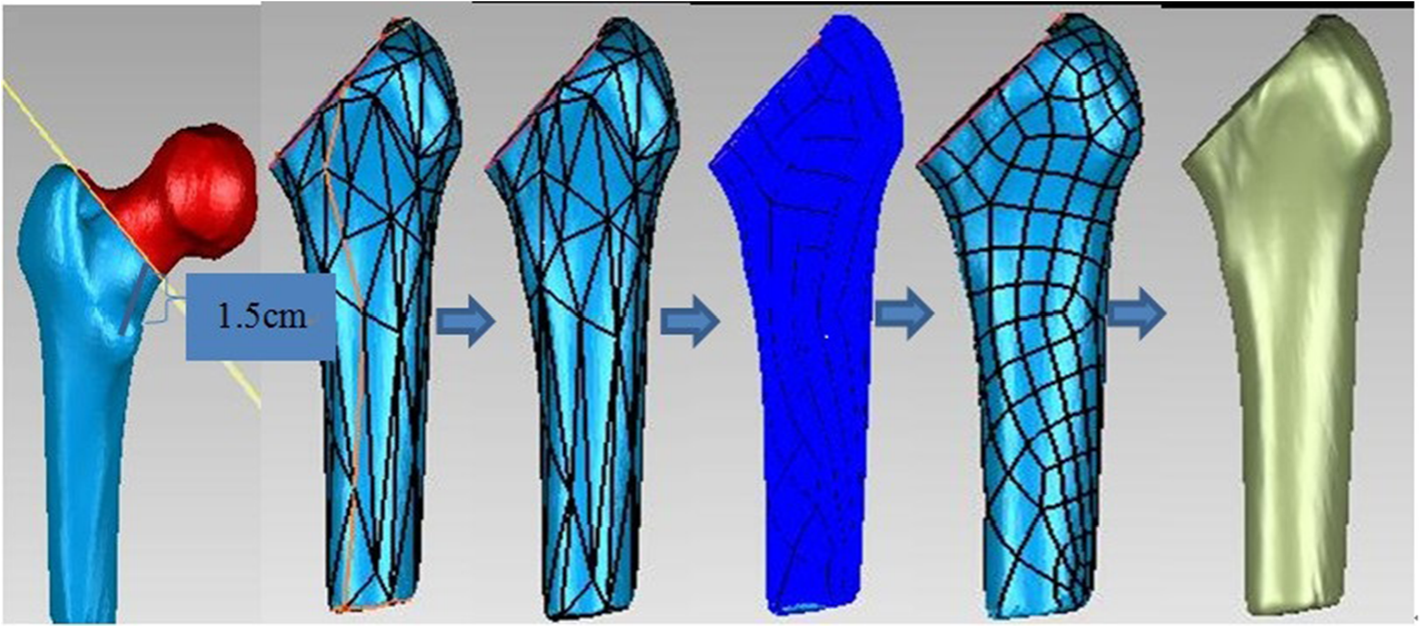
NURBS kyrtograph fitted for femoral shafts. **a** Simulative osteotomy. **b** Curvature detected. **c** Outline downgraded. **d** Patch constructed. **e** Grating constructed. **f** Kyrtograph fitting

The aforementioned 3D model was imported to Reverse Engineering software Geomagic Studio and then faired-up by the function of “Grid doctor”. The osteotomy along the base line of femoral neck was processed by “Plane section” module so that 1.5 cm medial femoral cortex is remained (Fig. 1a), which was performed through a series of procedures called “Probe curvature” (Fig. 1b) → “Degraded contour” (Fig. 1c) → “Construct surface patches” (Fig. 1d) → “Construct grid” (Fig. 1e) → “Fitting surface” (Fig. 1f) etc. A curve contour graph, called Non-uniform rational Basis spline (NURBS) cyrtography, was then fitted (Fig. 1).

#### Solid model fit

The above acquired cyrtograph models of the femoral shaft were exported to commercially available “Interactive CAD/CAM software”, UG-8.0, for further refinement. An entity model of solid was conversed by its functions of “Insert → Combine → Fit”, and clicking the “Checking Geometric Solid” module with the Surface Sweeping Method to check model until complete.

#### Femoral prostheses preparation

**Fig. 2:**
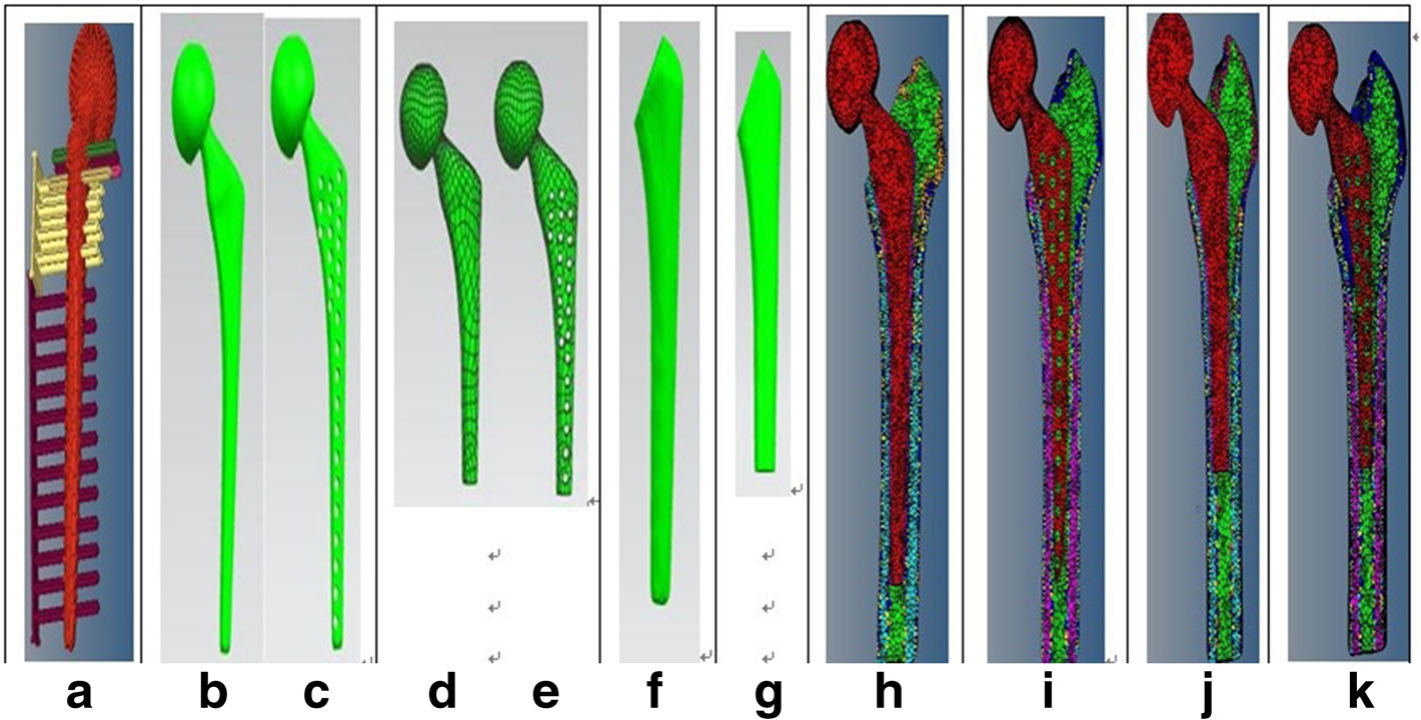
Prosthesis model & Punching-bear model. Cemented model & Sectional views of Grid model

1. A “Long-stem femoral prosthesis” of 135 mm was obtained (Fig. 2b) when patients with femoral head replacement underwent CT and were similarly processed as the aforementioned “***Distal femur developed***” session.
2. A “Long-stem femoral porous prosthesis”: a punching-bear with 27 cylinders of 4 mm diameter was created by Pro-E (Fig. 2a), and was then imported onto Hypermesh together with the (1) “Long-stem femoral prosthesis” so that this long-stem is punched to be “porous prosthesis” (Fig. 2c) through “Boolean calculation”.
3. A “Short-stem femoral prosthesis” of 100 mm (Fig. 2d) was developed after simulatively osteotomized by 35 mm from the (1) “Long-stem femoral prosthesis”.
4. A “Short-stem femoral porous prosthesis” with 23 cylinders that had diameters of 4 mm (Fig. 2e) was acquired after (3) “Short-stem femoral prosthesis” was punched with a “Boolean calculation”, similarly to procedure of item (2).

#### Osseous cement preparation

The total hip prostheses were amplified by 2 pixels during the Mimics execution and then extracted by *.stl format. Simulative osteotomy occurred on Geomagic-studio along the break-angle of prosthesis by same direction. The remaining prosthetic stem, which was amplified by 2 voxels, was imported onto UG to fit 3D-solid model. The models were then exported to Hypermesh to remove the original prosthetic stem from the amplified prosthetic stem by Boolean calculation. The virtual model of the bony cement layer with 3 mm-thickness [10] and a cavity are shown in Fig. 2f & 2g.

#### Assemblies of the replacement model

The above models of the femora, prostheses and cement were synthetized in Hypermesh to assemble the following four replacement types: (a) long-stem cemented prosthesis (Fig. 2h), (b) long-stem porous prosthesis (Fig. 2i), (c) short-stem cemented prosthesis (Fig. 2j), and (d) short-stem porous prosthesis (Fig. 2k).

#### Joint material assignment

A distal femur can be obtained after removing the space occupied by the prosthetic cemented stem or porous stem by Boolean calculation. The femora and other compartments were volume-meshed with cell-attribute assignment. All models of femora, prostheses and cement were exported back into Mimics for materials assignment. Then, the elastic modulus was automatically calculated. Based on CT gray-scale for bone, the elastic modulus was divided into five uniform scale so that five types of bony materials were assigned (with the minimum for inner and maximum for outer) according to a known formula [such as: Density *p* = 1017 × Grayvalu-scale - 13.4 (g/cm^3^), Modulus = 5925 × Density-388.8 (MPa)]. Poisson’s ratio was assumed to be 0.3. The properties of the remaining materials are based on prior studies [11, 12]. In this way, the geometrical 3D FE models of classic stems were obtained.

#### Loads configuration & boundary constraint

**Fig. 3:**
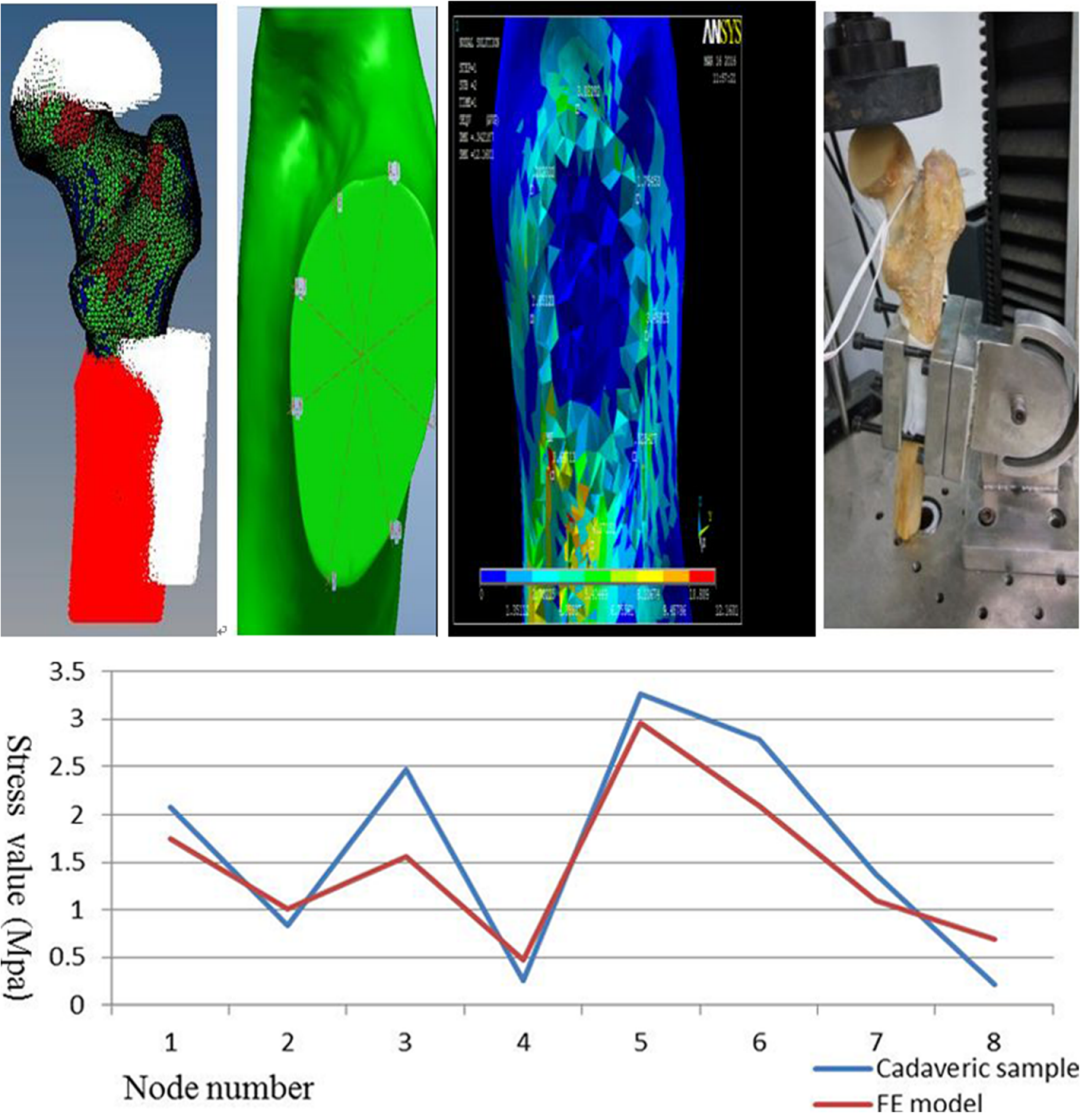
Verification of model validity. **a** Constrain. **b** Node plane. **c** Model validity. **d** Cadaveric validity. **e** Line chart for stress comparison between FE model and cadaveric femur

The above grid models are to be imported back to Hypermesh so that a compressive preload of 600 N was applied vertically and applied on the bone structures symmetrically. The degree of freedom on a node basis of *x-y-z* direction of Cartesian coordinate was constrained to 0, and translational displacements at the distal nodes of the femur were inhibited (Fig. 3a, 3b & 3c) [13, 14].

#### Model validation test

There are two ways to validate FE Models, which include the following: (1), Numerous model testing — To compare the present method with previous studies of similar stress / displacement, and (2), Cadaveric model testing — To develop a cadaveric model that resembles FEM in vitro. Its validity will be verified by comparing the similarity of outcomes for each experiment based on equal status.

It is assumed that FE model of the femoral neck is resected by a plane horizontally, and the circumference is intercrossed by four lines at 45° angles, forming 8 nodes (8 × 45° = 360°). When 600 N is vertically pressed on each of the corresponding 8 nodes of 12 samples [15] (these 12 samples are for model validation which are different from those 10 sets of spacemen for virtual surgery), the average values of stresses were acquired, as presented in Table 1. On the other hand, strain-foils were attached at relative positions of an intact cadaveric femur corresponding to FE model and mounted to UTM [12] (Fig. 3d). The load-strain curve was observed by recording the strain value when the maximal pressure of 600 N was reached. Force values were calculated to form Table 2 with these formulas: Actual-force = 10 × Measured-force - 6 (Mpa), Stress = Actual-force × Elastic-modulus (here, 7300 Mpa was chosen according to a reference [16]).

**Table 1:** Stress values for each 8-node of 12 FE samples

Position # Sample #	1	2	3	4	5	6	7	8
1	0.95962	1.08178	0.722216	0.222289	2.21429	1.231093	0.725866	0.600427
2	2.365501	0.522318	2.214457	0.290111	4.60191	2.907203	1.0395	0.713425
3	1.89497	1.204012	1.012684	0.467758	2.768523	2.102543	1.328077	0.819144
4	0.620409	0.399854	1.045387	0.371484	2.942854	2.261493	0.730747	0.462416
5	3.186124	1.094655	2.260299	0.609772	2.836237	2.868277	1.982035	0.202032
6	1.23152	2.22689	2.58363	0.754154	4.946323	2.627293	1.195486	0.950645
7	1.54106	1.06443	1.885397	0.354257	2.39623	1.369875	0.971287	0.798693
8	1.703297	0.650252	1.278543	0.205116	1.87754	1.337363	1.518337	1.046773
9	2.173445	1.507025	1.764093	0.862081	3.858323	3.472113	1.147653	0.367901
10	2.38251	0.916799	1.276729	0.970677	3.069833	2.331347	0.668171	0.883533
11	1.507317	0.710776	1.338166	0.412746	2.74588	1.366152	0.683243	0.581944
12	1.417896	0.720122	1.34875	0.19126	1.19263	1.19263	1.249671	0.896941
Average (MPa)	1.748639	1.008243	1.560863	0.475975	2.954214	2.088949	1.103339	0.693656

**Table 2:** Stress values for each 8-node of cadaveric femur

Node #	Strain #1	Strain #2	Strain #3	Strain average	Absolute value	Stress (MPa)
1	250	290	315	285	285	2.0805
2	−110	−120		−115	115	0.8395
3	−325	−350		−337.5	337.5	2.46375
4	−35	−35	−35	−35	35	0.2555
5	425	455	460	446.6667	446.7	3.26091
6	380	385		382.5	382.5	2.79225
7	190	185		187.5	187.5	1.36875
8	−30	−30	−30	−30	30	0.219

#### Finite element solution

This generated FE model was exported onto AnSys package for computation and analysis, and the peak and distribution yield were measured. The observation index includes (1) Von Mises stress / displacement contours of femur / cement / prosthesis, and (2) Stress / displacement contours of the general model.

## Results

### Observations during model testing

#### Numerous model validations

The current testing outcome was consistent with the validation technique and study conducted by Zhang et al. [17], the comparability of the current result and previous results indicates that our FE model is valid.

#### Cadaveric model validations

Tables 1 and 2 can be consolidated into Table 3 and are interpreted in Fig. 3e. A significance of *p* > 0.05 from the Independent Sample Test indicates that in vitro cadaveric testing and the FE models are not significantly different (they are agreeable to each other), which validates our approach for further simulation and analysis.

**Table 3:** Stress comparison on correspond positions of FE model to cadaveric femur

Node # Sample #	1	2	3	4	5	6	7	8
FE model	1.7486	1.0082	1.5609	0.4760	2.9542	2.0889	1.1033	0.6937
Cadaver	2.0805	0.8395	2.4638	0.2555	3.2609	2.7923	1.3688	0.2190

### Observation of stress distribution

#### Stress of prostheses

**Fig. 4:**
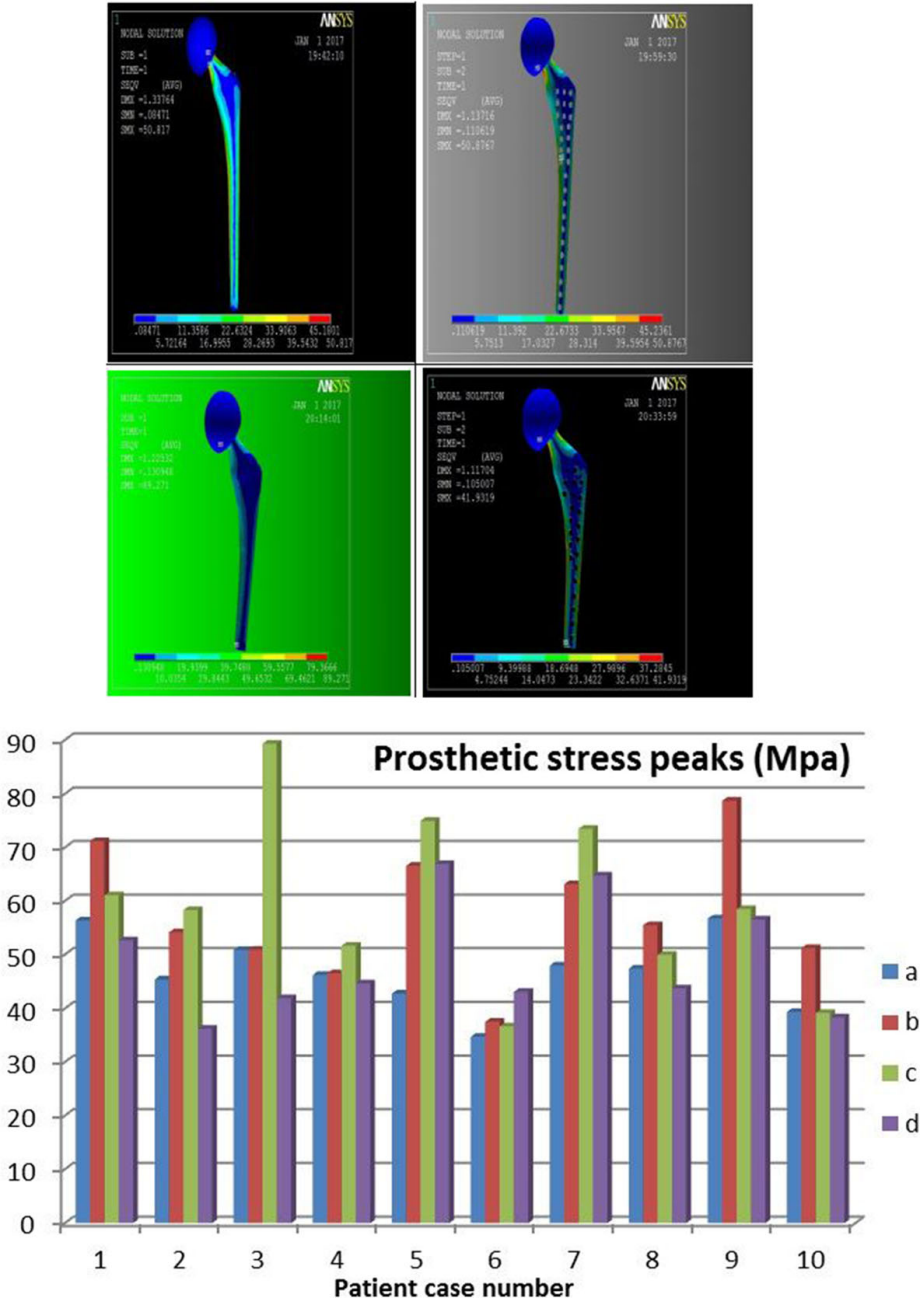
Stress distribution of femoral prostheses & Histogram of femoral prosthetic stresses

As depicted by Fig. 4, generally, the contact stresses of all femoral-head prostheses are concentrated around prosthetic stems equably. For model (a), the pressure crest value is across the edge of femoral-head intersecting the prosthetic stem. For style (b), the summit value is along the middle of prosthetic stem. For style (c), the stress peak appears at the intersection of prosthetic terminus and bony cement. For style (d), the stress apex is located at prosthetic head crossing neck as well as the middle stem. The prostheses are easier to damage from these locations [18].

As depicted by Table 4, the mean stress value of each model is as follows: (a) = 46.7682, (b) = 57.5332, (c) = 57.2927, and (d) = 48.8891 (MPa). The variance homogeneity test and analysis demonstrates that these four types are indifferent significantly (*p* = 0.064 > 0.05) according to SPSS-13, which could be simplified as (a) ≈ (b) ≈ (c) ≈ (d). However, all of these models meet the mechanical prerequisite of femoral-head replacement because the maximum stress of each model is less than the Yield Strength of the titanium alloy, which is 600 ~ 900 MPa.

**Table 4:**
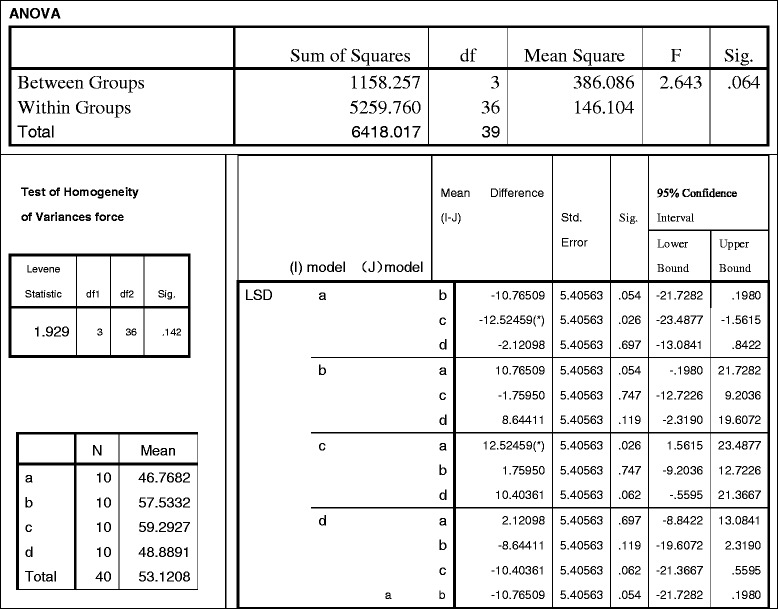
Statistics of prosthetic stresses peak (variance homogeneity test & analysis)

#### Stress on femora

By reviewing the Von Mises variation of FEA data, the reaction stresses of femora are distributing equally inferior to femoral trochanter and superior to the constrained position. Additionally, stress peaks disperse around the interface of the prostheses terminus and femur.

When observed within Table 5, the stress mean-value of each model is as follows: (a) = 10.6316, (b) = 9.5798, (c) = 17.4404, and (d) = 9.5140 (MPa), which are less than the Yield Strength of femur (104 ~ 120 MPa) according to previously published studies [19]. Based on the factor of variance, all were *p* < 0.001 < 0.05, which suggests the four styles were significantly different. When analyzed by pairwise comparison between the other three models to determine the biggest stress (c), *p* < 0.05 indicated that the cemented short-stem (c) was the most unsuitable type for hip joint implantation [shortened by (d) < (b) < (a) < (c)]. When further analyzed by pairwise comparison among (a) (b) & (d), *p* > 0.05 implies that the porous short-stem design (d) was not significantly different from the long-stem (b), and (d) was also indifferent from cemented long-stem (a) significantly, which can be simplified as d (d) ≈ (b) ≈ (a) < (c).

**Table 5:**
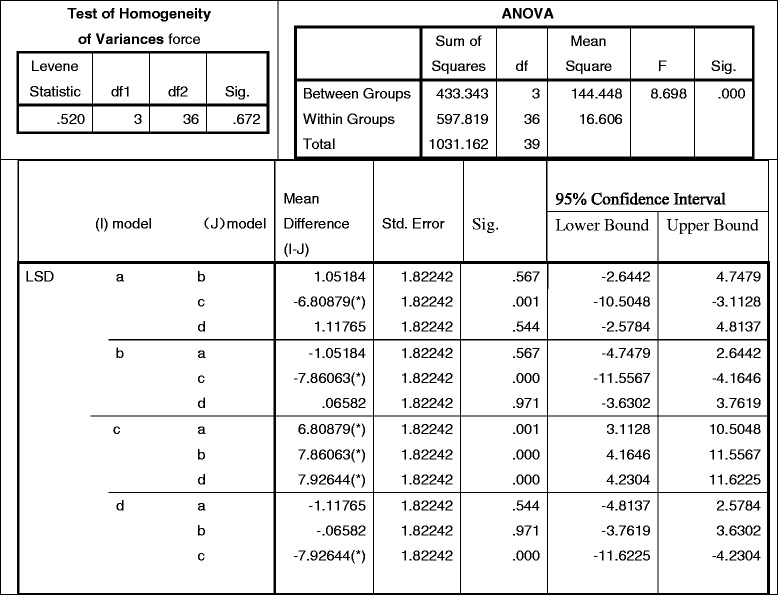
Statistics of femoral stresses peak (pairwise comparison among 4 models)

#### Stress on integral joints

Based on the Von Mises outcome, the contact stresses of any type of integral joint distributed similarly as the prostheses, the stresses peaked on the prostheses and decreased from the maximum value at the femoral fixation point to the minimum values at the proximal end. By reviewing Table 6, the stress mean-value of each model is as follows: (a) = 47.4895, (b) = 58.5053, (c) = 60.4703, and (d) = 45.8822 (MPa). These values were analyzed by a factor of variance: all *p* = 0.031 < 0.05, suggesting a significant discrepancy [simply described by (d) < (a) < (b) < (c)]. When analyzed by pairwise comparison, *p* < 0.05 indicates they are significantly different, but *p* > 0.05 suggests the difference between (a) v.s. (d) was not significant statistically, which is (d) ≈ (a) < (b) < (c). Mechanically, the (a) cemented long-stem and (d) porous short-stem are the least stressed.

**Table 6:**
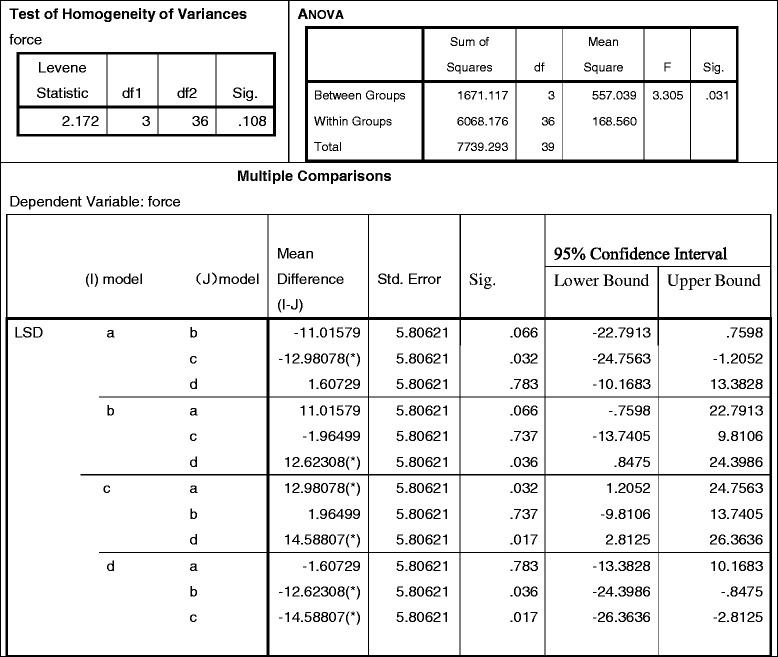
Statistics of integral joint stresses peak (pairwise comparison among 4 models)

### Observation of displacement distribution

#### Displacement of femora

**Fig. 5:**
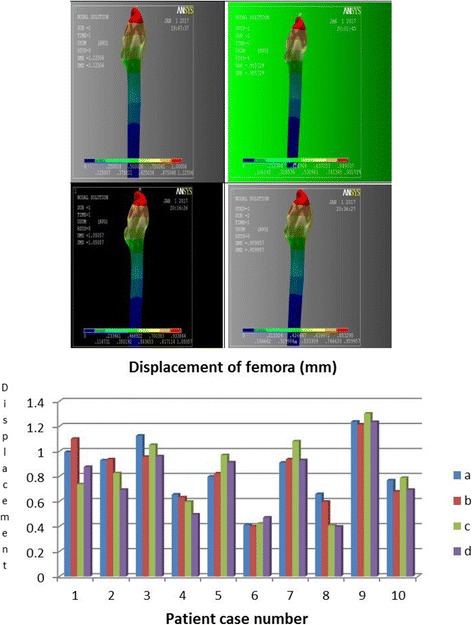
Von Mises of displacement for femurs & Femoral displacement Histogram

Femur displacement implies its magnitude of stability relative to femoral joint. Through observation of Fig. 5, we found that peak displacement of all models was concentrated near the greater-trochanter adjacent region, which decreases distally in concentric circles. The direction of migration was vertical downward. The displacement mean-value of each model is as follows: (a) = 0.8484, (b) = 0.8269, (c) = 0.8179, and (d) = 0.7658 (mm); a significance of *p* > 0.05 when analyzed by pairwise comparison suggests the difference is not significant.

#### Displacement of integral joint

The displacement of integral joint implies its magnitude of fastness relative to the entire femoral joint. By observation of finite element data, we found that the direction of displacement was vertically downwards, the all peak displacements were concentrated on the head of prostheses and decrease distally in concentric circles. The displacement mean-value of each model was as follows: (a) = 1.0257, (b) = 0.9984, (c) = 0.9915, and (d) = 0.9849 (mm), a significance of *p* > 0.05 when analyzed by pairwise comparison suggested the risk of coxa adducta among all joint types was significantly indifferent.

#### Displacement of prostheses

The distribution of prosthetic displacement behaves similarly to that of the integral joint.

## Discussion

Artificial short stems for uncemented total HA have been alternatively practiced to conventional stem designs. However, there is little biomechanical examination for effects of stem length and stem character on surgical complications [20]. To isolate clinical variables, the theoretic test performed by FEA is essential to Mimic ultimate prosthetic geometry prior to actual fabrication because it is rectified to fail practically if a stem fails at this stage. Computational FEM has been extensively employed in predicting optimized implants prescribed by surgeons [21]. The strength of FEA carried out by computer aided simulation enables preoperative strategy and decreases procedure time. This experiment of “outside bone and outside body” is also harmless for humans or animals. Our models were not only consistent with predecessors, and they were also similar to cadaveric data. These punched prostheses were designed virtually with mechanic compatibility and bio-compatibility for clinical application. These structures may induce bony ingrowth at the porous interface, which is beneficial to bioactivity. Additionally, all the four femoral stems were anatomically tailored to individualize the femoral structure towards stress and displacement.

Displacement is an indicator for determining the stability of newly designed stems. Several factors such as implant shape, stem size, bone-implant gap are accounted for micromotion [22]. Regarding cemented stems, fastness between bone and cement relies on penetration of the cement upon cortex. The mechanical interlocking between these two constituents is correlated with bony stress surrounding the cement. A Load-transfer mechanism takes place at this interface, and cementing mechanization is driven by friction rather than adhesive properties [23]. Cemented prostheses (a) / (c) achieve primary stability mechanically through locks between cement surrounding the bone and implant, which inhibits distal migration by osseointegration. Cementless prostheses (b) / (d) induce bony ingrowth onto the implant surface, which is biologically referred to as secondary stability. Previous studies indicate that the amount of ongrowth is inversely proportional to the amount of micromotion [24]. With respect to porous stems, Manley et al. reported that uncemented porous prostheses allow for bone ingrowth to achieve rigid fixations, but the shortcoming of osteolysis influences their long-term stability [25]. These results conflict with Ellison’s view that the “porous stem encourages stable fixation and securely seals joint space by preventing migration” [26]. At this time, we numerically demonstrated the degree of stabilization using the analytical FE approach. When double tested statistically by SPSS, *p* > 0.05 for displacements of (a) / (b) / (c) / (d) implied that these four sorts of stems attain fastened equally after installation.

Pre-stress to bones can be created in femoral canal by internal fixation, which is probably susceptible to failure based on the stresses distribution onto the modular junctions of the implants. Suppose the joint with implant is loaded in equivalency. In terms of contact stresses upon femoral-head prostheses, the statistical dataset [(d) ≈ (a) ≈ (b) ≈ (c) in simplification] demonstrates these four types behave similarly in quantitation; In terms of the stresses upon femurs, the statistical data in order of (d) ≈ (a) ≈ (b) < (c) demonstrates that type (c), the “Cemented short-stem”, performs the worst; In terms of the stresses on the whole joints, the statistical data supporting (d) ≈ (a) < (b) < (c) demonstrates that type (b), “Porous long-stem”, is also not a good option. In sequence, the least stress (d) ≈ (a) supports that type (a) “Cemented long-stem” and (d) “Porous short-stem” distribute optimal loads and can be used in practice. There are several possible mechanical reasons for these outcomes: (1), If designed with cemented, when considering the stem, because quota-stress is shared by wider area when length is extended, stress on long-stem is less than short-stem. When considering the cement, which is the weakest material and has a higher risk of fatigue failure at the cement-bone interface, maximum stress on the cement increases when the length increases, a longer distance between the loading-point and fixation-point induces larger bending-stress at the distal end. (2), If designed with uncemented, long-stem appears to be superior to short-stem by the reasoning in (1), but this statement is actually controversial as follows: short-stems enable sufficient space for complete osseous ingrowth, preserving greater stability, this design also confers higher resistance from torsional forces and has a lower risk of peri-prosthetic fracture than long-stems. With respect to the survival rate (99 ~ 100% for 10 years) reported by McLaughlin et al. [23, 27], short stem designs are suggested as alternative to longer stem designs. (3), If designed with “punched”, porous stems provide meaningful structural support. The porous nature enables bony ingrowth, and the new bones can handle load and enlarge surface area [28]. As a result, stress on cementless-stem with pore is sequentially less than cemented-stem without porous coating [4, 29]. Therefore, short-stemmed punched-prostheses are generally thought to facilitate surgery compared to long-stemmed cemented-prostheses.

However, “the least” doesn’t mean “the best” and “minimal” isn’t equal to “optimal”, because minimal stress doesn’t guarantee the best choice for a clinical trial. The prostheses inserted into the femoral cavity might change the normal stresses distributions. Stress can be spread to the distal femur via intramedullary prosthesis despite its support by the proximal femur, which leads to stress-shielding, affecting the osseous integrity and resistance [30]. Longer stems are believed to give higher stresses at the femur and then be capable of reducing stress shielding problems, whereas shorter stems decrease the load-transfer to the cortical bone and encourages stress shielding and bone resorption, which results in failure of the prosthesis sometimes [31]. Chen WP et al. revealed that less stress shielding occurs in those femurs with fully cemented fixation [32]. For an uncemented short stem, even if Maier reported it is curved stems but not straight stems that cause cortical hypertrophy, there is no significant effect on the clinical outcome at early follow-up [33]. Regarding porous prostheses, Ellison et al. provides evidence that these prostheses do not cause stress shielding in 14-year follow-up [26]. As another result, the module that achieves the minimum stress-shielding is the cementless prosthesis, due to poor results of cemented components after revision surgery, short-stem of porously punched uncemented prosthesis has become more popular.

## Conclusion

In summary, this study proposes long v.s. short stems of cementless v.s. cement in 3D models providing statistical analysis in comparison with conventional methods. The data support the hypothesis that stresses and distributions of prosthetic stems are various from types or sizes: Mechanically, displacement outcome has equal fastness for these four types of femoral stems, and the mechanic result is positive for both cemented long-stem and uncemented short-stem designs. Clinically, the cemented long-stem design is suitable for patients with osteoporosis, while the porously punched cementless short-stem is preferable for those with easy osseo-ingrowth or with cement allergy. Functionally, the advantage of the punched-prosthesis designed by virtual computation without a cement matrix is that it could be immobilized by the flesh bone structure growing inside the prosthesis orifices. Not only is the contract stress reduced, complications in cement are also avoidable, and this approach should be recommended as reasonable alternative for femoral-head replacement.

## Abbreviations

3D: Three Dimensional
CAD: Computer Aided Design
CAM: Computer Aided Manufacturing
CT: Computed Tomography
FE: Finite Element
FEA: Finite Element Analysis
HA: Hip Arthroplasty
THA: Total Hip Arthroplasty
